# Evaluation of wet cupping therapy on the arterial and venous blood parameters in healthy Arabian horses

**DOI:** 10.14202/vetworld.2018.620-626

**Published:** 2018-05-14

**Authors:** Turke Shawaf, Wael El-Deeb, Jamal Hussen, Mahmoud Hendi, Shahab Al-Bulushi

**Affiliations:** 1 Department of Clinical Studies, College of Veterinary Medicine, King Faisal University, 400 Al-Hasa, 31982, Saudi Arabia; 2 Department of Veterinary Medicine, Infectious Diseases and Fish Diseases, Faculty of Veterinary Medicine, Mansoura University, Mansoura, Egypt; 3 Immunology Unit, University of Veterinary Medicine Hannover, Foundation, 30173 Hannover, Germany; 4 Department of Microbiology and Parasitology, College of Veterinary Medicine, King Faisal University, 400 AlHasa, 31982, Saudi Arabia; 5 Veterinary Al-Waha Clinic, Qatar Street, Hofof, AlHasa, 31982, Saudi Arabia

**Keywords:** biochemical, cortisol, cupping, hematological, horse

## Abstract

**Aim:**

Recently, the complementary therapies such as cupping and acupuncture are being used in veterinary medicine. This research was carried out to determine the effects of wet cupping therapy (Hijama) on the hematological and the biochemical parameters in the healthy Arabian horses for the first time.

**Materials and Methods:**

In this study, seven clinically healthy Arabian horses were randomly selected. Four points on the animal body were selected to perform the cupping therapy. Two points were selected at the back just behind the scapula on the left and right sides; another two points were located in the rump. Cups with 4 oz (125 ml) size with narrow mouths were used. A manual pump (sucking cups) was used to create the negative pressure within the cups during cupping. Arterial and venous blood parameters and serum cortisol concentration were measured before cupping and 3 days and 2, 4, and 8 weeks after cupping.

**Results:**

No significant differences were estimated in most hematological and biochemical parameters after cupping. A significant decrease in the concentration of serum cortisol was observed in 3 and 14 days after cupping.

**Conclusions:**

Cupping induced minor changes on the hematological and biochemical parameters in Arabian horses. This is the first trial on the effects of wet cupping therapy on the different parameters in Arabian horses, which would be useful for further investigations on the role of complementary therapies in horses. Our further studies will include different disease models.

## Introduction

Cupping is the process of drawing blood from the body by the scarification and the application of a cupping glass. Cupping therapy is considered as one of the conventional methods of treatment that are commonly used in Asian and Middle East countries [1,2]. Some resources mentioned that cupping has been used in some countries such as Egypt and China since more than 2000 years [3,4]. Cupping is also known as Baguan in China in which Ba is referred to pulling up and guan means the cups that are used [5]. The cupping process involves using a cup and a suction system that is used to remove the air that is present in the cup which causes negative pressure inside the cup that draws the skin and the subcutaneous tissues in it [5]. There are two main types of cupping, which are dry and wet cupping therapy [6]. In dry cupping, the suckling effect is created by using heated cups or an air pump to siphon the air out of the cup, which creates a vacuum that tugs the skin upward. The wet cupping therapy is considered as the most commonly used method. It is performed by placing a glass cup on the skin, and the skin is pulled in it through creating a vacuum inside the cup. A superficial incision is made in the same area after few minutes, and the blood will be allowed to accumulate inside the cups. This process can be repeated multiple time [7]. Hijama is considered as one of the wet-cupping therapies types in which this type is widely practiced in the Muslims countries [8]. This method can be applied to many regions such as the back of the neck, chest, abdominal wall, and the back area [2]. This type of therapy is used to relieve the pain, improve body metabolisms such as digestion, to remove bad blood from the body, and for some of the conditions such as flu, heatstroke, asthma, bronchitis, and musculoskeletal problem [1,2,9,10]. The cupping therapy can be used alone or in combination with some other traditional treatment methods such as acupuncture [2]. Cupping could be an uncomfortable process, which may need animal sedation. In addition, wet cupping could cause skin irritation or infection if the site of the treatment was not well cleaned before and after cupping procedure.

Cupping therapy is contraindicated to be used over the regions that have injured flesh or dermis, abrasions, sunburns, and contusion; further, heavy and long cupping therapy is contraindicated to be used with the patients that are undertaking anticoagulants courses [5]. Cortisol concentration in equines has been used to determine stress generated by illness, pain, and lack of welfare [11]. Cortisol has gained interest due to its prognostic value in the critical phases and also as an indicator of fitness and athletic performance in sports horses [12]. In these days, the complementary therapies such as cupping and acupuncture are being used in veterinary medicines.

Insufficient information is known about the effect of cupping on animals. Therefore, this research was carried out to determine the effects of wet cupping therapy on some hematological and the biochemical parameters in healthy horses.

## Materials and Methods

### Ethical approval

This investigation was approved to be carried out by the Ethics Committee at King Faisal University in Saudi Arabia for research purposes.

### Animals

To conduct this study, seven clinically healthy Arabian horses (five Mares and two Stallions) from the Agricultural and Veterinary Research Station, King Faisal University, Saudi Arabia, were randomly selected. Their body weight ranged from 370 to 480 kg, and their age was from 4 to 14 years. This study was carried out during April and May 2017, and the cupping procedure and all blood samples were collected from fasting animals between 9:00 and 11:00 AM. All horses were given unified feed mixtures that include grains (corn, barley, and oat), concentrate cubes, and hay without any feed additives. It is well accepted that establishment of the new procedure begins with experiments on healthy animals. We, therefore, decided to use healthy horses in our study. However, further studies will include different disease models.

### Cupping technique

#### Identification of the cupping area

**Figure-1:**
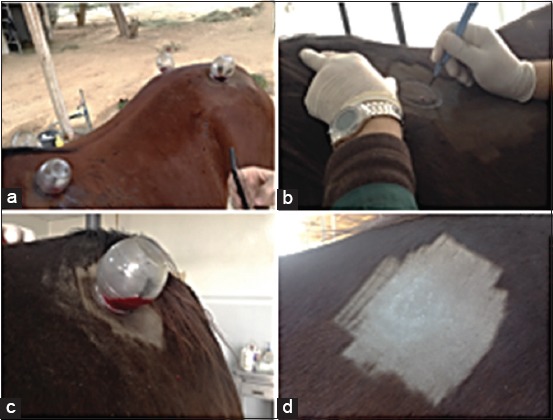
(a) Image showing the position of cupping in horses two places located behind the shoulder (left and right) and the other two places located on the rump (left and right) in the level of sacrum; (b) Uses a blade to make some superficial incisions on cupping areas; (c) Bleeding in progress into the glass cups after incising the skin with a blade. (C and d) Place of wet cupping therapy (7 days after cupping procedure).

Due to lack of published information about the application of cupping in horses, we had adopted the meridian of energy in traditional Chinese medicine and to identify some of the points that happen by the pool of blood in the body of the horse avoiding the application of the cups over an artery, deep-vein thrombosis, or ulcer. Four points in the animal body were selected to perform the cupping therapy. Two points were selected at the back just behind the scapula on the left and right sides; another two points were located in the rump (bounds of sacral vertebrates) as shown in Figure-1a.

#### Cupping procedure

The cupping procedure was applied to the horses in the morning (9 AM). After the determination of the cupping area, the areas were washed with soap and water and shaved to remove the body hair. Moderate amount of olive oil was applied to the relevant area; this step can make the reposition of the cups much easier as needed during cupping and may be considered as a seal between the mouth of the cup and the skin. The size of the cups that were used was 4 oz (125 ml) with narrow mouths. Cups were applied with a hand-held, manual pump (sucking cups) that is used to create the negative pressure within the cups during cupping. The negative pressure in the cups was formed through heating the air that is found inside the cup to make more vacuum pressure in cups during cupping therapy. The air was heated by wiping the internal surface of the cups by swabs that contain alcohol then flame it [13]. After that, a blade was used to make some superficial incisions on the same areas (Figure-1b). Once these incisions have been made, the cups are then re-placed and left in the area for up to 10 min to suck the blood and allow it to accumulate in the cups (Figure-1c). The cup was removed after it was completely filled and another cup was placed on the same area to suck more blood. This had been repeated for three times. Approximately, 200 ml of cupping blood was collected from each animal. No complications, such as skin irritation or infection, were observed on the areas of cupping for 8 weeks after cupping procedures (Figure-1d).

### Blood sampling

#### Venous and cupping blood

Venous blood samples were obtained from the horses through jugular vein puncture before cupping and 3 days and 2, 4 and 8 weeks after cupping into two vacutainer tubes (Guangzhou Improve Medical, China), in which one of them contained ethylenediaminetetraacetic acid for the hematology and the second tube was free from any anticoagulants for the biochemical analysis of selected parameters. The blood was allowed to clot, and after centrifugation, the serum was separated and stored at −20°C until it was analyzed. Cupping blood was collected at the cupping time by absorption from the cupping region into two tubes as prescribed for the venous blood. Cupping blood collected in the second cup that was applicated on the left shoulder region of the animal was used for these investigations.

#### Arterial blood

For blood gas analysis, arterial blood was collected from a common carotid artery in a heparinized syringe using 20G injection needle into capillary glass tubes containing heparin before and 14 days after the cupping. After blood collection, the syringes were kept capped on crushed ice to be analyzed for the blood gases within 5 min.

### Blood gas analysis

Arterial blood samples were analyzed using a 248 pH/Blood Gas Analyzer for the assessment of the partial pressure of oxygen (PaO2), partial pressure of carbon dioxide (PaCO_2_), HCO_3_, potassium (K), sodium (Na), calcium (Ca), and pH. Rectal temperature was measured by a thermometer for correction of gas tensions by the analyzer.

### Hematological analysis of blood samples

Venous blood and the cupping blood hematology were carried out using CELL-DYN 3700 analyzer within 45 min after blood collection for the total red blood cells count (RBC), total and differential white blood cells count (WBC), hematocrit (HCT), mean corpuscular volume (MCV), mean platelet volume, hemoglobin (HGB), mean corpuscular HGB (MCH), MCH concentration, red cell distribution width (RDW), procalcitonin (PCT), and platelets (PLT).

### Serum biochemical analysis

The biochemical analysis of cupping and venous blood was carried out from the serum within 24 h. Albumin (ALB), aspartate aminotransferase (AST), alkaline phosphatase (ALP), gamma-glutamyl transferase (GGT), total protein (TP), globulin (GLOB), blood urea nitrogen, creatine kinase (CK), Ca, Na, K, glucose (Glu), total bilirubin (TBIL), and creatinine (CRE) estimation were carried out using Vet scan versus 2 analyzer (ABAXIS, USA).

### Determination of the serum cortisol concentration

Cortisol levels in the serum samples were estimated by enzyme-linked immunosorbent assay using a kit that is available commercially for cortisol (DEMEDITEC, Kiel, Germany).

### Statistical analysis

The obtained results were analyzed using GraphPad Prism 7 in which the range, mean, and standard error of the mean were determined. The significance between results were determined using Student’s t-test in which the results considered significant when p<0.05, and the normal distribution of values were evaluated using D’Agostino and Pearson omnibus normality test.

## Results

**Figure-2:**
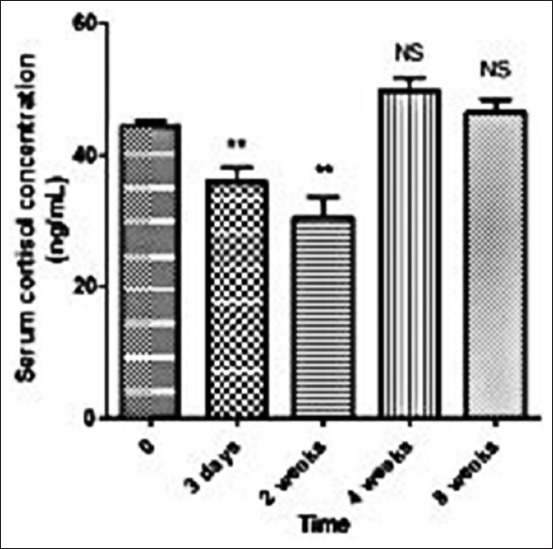
Variation in cortisol levels during a time unit.

The obtained results of the hematological and biochemical analysis before and after the wet cupping therapy (Hijama) are summarized in Tables-1-4 and in Figure-2. During the period of blood samples collection (8 weeks after cupping), information were obtained from the horse’s breeder about the condition of the horses after the cupping, in which the observer observed improvements in the general condition of the horses. Mares also showed more clear indications of fertility cycle. No statistically significant differences were observed in most of the venous blood hematological and biochemical parameters (Tables-1 and 2). However, there were significant differences between the venous blood and the cupping blood samples in their WBCs, lymphocytes (LYM), basophils (BASO), RDW, PLT, ALP, GGT, GLOB, CK, TBIL, CRE, and Na as shown in Table-3. Significant decrease in WBCs and BASO with increase in LYM, RDW, and PLT was observed in the cupping samples when compared to the venous samples. The differences in the blood cell composition between venous blood and cupping blood may be due to selective migration of defined blood cell populations into the site of cupping. For the biochemical analysis, significant increase in ALP and CK levels and decrease in GGT, GLOB, TBIL, CRE, and Na values were observed in cupping blood samples than their concentrations in the venous blood samples. Non-significant changes were observed in PCT values. As shown in Table-2, a significant increase in TP was detected after 2 and 4 weeks of cupping in all horses. In the same concern, GLOB and Glu levels were significantly increased 3 days and up to 8 weeks after cupping. In contrast, significant decrease was observed in Na, CRE, and TBIL in the period between 2 and 8 weeks post-cupping. The results that are listed in Table-4 show a significant decrease of pCO_2_ and a significant increase of pO_2_ which were observed in 2 weeks after cupping compared to their levels before the cupping. However, the pH decreased non-significantly in 14 days after applying the cupping therapy compared to its value before the cupping. The levels of serum cortisol were significantly decreased in horses in 3 and 14 days after cupping when compared with their levels before cupping, while showed non-significant increase in their levels in the period between 4 and 8 weeks after the cupping therapy when compared to their levels before the cupping as shown in Figure-2.

**Table-1:** Mean and SEM of the hematological parameters in Arabian horses before and after cupping.

Parameter	Before cupping	After 3 days	After 2 weeks	After 4 weeks	After 8 weeks
WBC (×103/mL)	8.05±0.13	7.18±0.56*	9.13±0.85*	9.87±0.42*	8.68±0.53
LYM %	31.42±4.13	23.48±4.79*	43.46±4.51*	42.7±4.64*	28.4±4.57
MONO %	3±1.03	2.28±0.92	4.84±0.8	3.3±0.1	0.6±0
NEU %	62.38±3.74	60.22±4.03	48.62±3.84*	50.12±3.96	67.82±4.96
EOS %	2.68±0.42	3.54±0.54	2.58±0.44	3.34±0.85	2.96±0.5
BASO %	0.48±0.09	0.5±0.15	0.52±0.13	0.6±0.17	0.22±0.08
RBC (×106/mL)	8.28±0.35	6.23±0.29*	8.37±0.3	8.27±0.61	8.86±0.53
HGB (g/dL)	14.48±0.55	12.08±0.49	15.48±0.65	15.04±0.87	16.58±0.68
HCT %	39.5±1.36	32.33±1.13*	40.1±1.65	40.11±3.11	42.27±1.95
MCV (fL)	47.8±0.73	47.4±0.75	47.6±0.81	48.4±1.03	48±0.77
MCH (pg)	17.5±0.21	19±0.31	18.56±0.7	18.3±0.42	18.04±0.32
MCHC (g/dL)	36.64±0.22	39.86±0.27	38.68±1.14	38.13±1.31	37.76±0.5
RDW %	22.42±0.37	22.38±0.29	22.82±0.3	23.62±0.14	22.8±0.18
PLT	152±16.12	119.2±22.44	179.4±40.45	160.4±31.47	85.4±14.02
PCT	0.10±0.01	0.086±0.02	0.138±0.04	0.14±0.02	0.05±0.01
MPV	6.96±0.18	7.08±0.18	7.3±0.45	7.08±0.12	6.88±0.34
RDWc	35.12±0.81	33.54±0.47	36±1.56	35.92±0.7	35.56±1.14

NS>0.05 and

* <0.05, SEM=Standard error of the mean, WBC=White blood cells, LYM=Lymphocytes, MONO=Monocytes, NEU=Neutrophils, EOS=Eosinophils, BASO=Basophils, RBC=Red blood cells, HGB=Hemoglobin, HCT=Hematocrit, MCV=Mean corpuscular volume, MCH=Mean corpuscular hemoglobin, MCHC=Mean corpuscular hemoglobin concentration, RDW=Red cell distribution width, PLT=Platelets, PCT=Procalcitonin, MPV=Mean platelet volume

**Table-2:** Mean and SEM of the serum biochemical parameters in Arabian horses before and after cupping.

Parameter	Before cupping	After 3 days	After 2 weeks	After 4 weeks	After 8 weeks
ALB (g/dl)	3.95±0.05	3.75±0.45	3.95±0.25	4.15±0.15	3.8±0.1
ALP (IU/l)	131.5±3.98	133.5±8.1	137±7.51	149.2±11.25	162.3±4.52
AST (IU/l)	239±94	335.2±24.3	270.4±37.8	278.3±18.7	235.7±22.8
GGT (IU/l)	12.1±2.79	13.4±2.05	12.5±2.13	14.85±1.96	12.8±1.52
TP (g/dl)	6.37±0.35	6.90±0.75	7.87±1.15*	7.90±1.85*	7.22±1.25
GLOB (g/dl)	2.42±0.35	3.15±0.45*	3.92±0.75**	3.75±0.1**	3.44±0.55*
BUN (mg/dl)	14.15±3.55	14.4±2.29	13.95±1.85	17.85±1.35	18.05±0.95
CK (IU/l)	129.95±47	96.2±12.6	90.1±16.8	133.8±14.8	91.8±24.4
Ca (mmol/l)	11.05±0.35	9.5±0.30	11.75±0.95	13.65±0.32	12.8±0.30
Na (mmol/l)	142.5±4.2	133.2±5.1*	132.5±9.4*	141.7±4.55	141.5±1.5
K (mmol/l)	4.42±0.4	4.12±0.49	3.75±0.7*	4.45±0.64	4.32±0.60
Glu (mg/dl)	90.3±10.2	88.5±5.5	110.4±4.2**	114±6.0**	94.2±1.1
TBIL (mmol/l)	2.21±0.73	2.2±0.21	1.65±0.35*	1.1±0.50*	1.85±0.35
CRE (mg/dl)	1.85±0.05	1.35±0.25	1.1±0.12**	1.05±0.15*	1.22±0.35*

NS>0.05,

* <0.05 and

** <0.01, SEM=Standard error of the mean, ALB=Albumin, ALP=Alkaline phosphatase, AST=Aspartate aminotransferase, GGT=Gamma glutamyl transferase, TP=Total protein, GLO=Globulin, BUN=Blood urea nitrogen, CK=Creatine kinase, Ca=Calcium, K=Potassium, Na=Sodium, Glu=glucose, TBIL=Total bilirubin, CRE=Creatinine

**Table-3:** Comparison between the hematological and biochemical parameters of venous and cupping blood samples of examined horses.

Parameter	Venous blood	Cupping blood	Parameter	Vein blood	Cupping blood
WBC (×103/mL)	8.05±0.13	3.15±0.052**	ALB (g/dl)	3.95±0.005	3.10±0.4
LYM %	31.42±4.13	39.10±3.98*	ALP (IU/l)	131.5±3.98	144.50±29.5*
MONO %	3±1.03	2.80±1.25	AST (IU/l)	239±94	210.50±18.5
NEU %	62.38±3.74	54.12±3.79	GGT (IU/l)	12.1±2.79	8.1±1.2*
EOS %	2.68±0.42	3.92±1.29	TP (g/dl)	6.37±0.35	5.20±0.90
BASO %	0.48±0.09	0.12±0.05**	GLOB (g/dl)	2.42±0.35	2.1±0.51*
RBC (×10^6^/mL)	8.28±0.35	8.42±0.80	BUN (mg/dl)	14.15±3.55	12.85±3.15
HGB (g/dL)	14.48±0.55	12.95±1.01	CK (IU/l)	129.95±47	333±41.2**
HCT %	39.5±1.36	33.73±2.27	TBIL (mmol/l)	2.21±0.73	1.45±0.55*
MCV (fL)	47.8±0.73	46.95±0.74	CRE (mg/dl)	1.85±0.05	1.0±0.03*
MCH (pg)	17.5±0.21	15.4±1.15	Ca (mmol/l)	11.05±0.35	10.10±0.71
MCHC (g/dL)	36.64±0.22	35.2±0.93	Na (mmol/l)	142.5±4.2	120.50±5.5**
RDW %	22.42±0.37	27.05±1.21*	K (mmol/l)	4.42±0.4	4.52±0.38
PLT	152±16.12	162.8±18.12*	Glu (mg/dl)	90.3±10.2	81.5±15.30
PCT	0.10±0.01	0.11±0.02			
MPV	6.96±0.18	7.05±0.14			
RDWc	35.12±0.81	34.97±0.56			

NS>0.05,

* <0.05 and

** <0.01, WBC=White blood cells, LYM=Lymphocytes, MONO=Monocytes, NEU=Neutrophils, EOS=Eosinophils, BASO=Basophils, RBC=Red blood cells, HGB=Hemoglobin, HCT=Hematocrit, MCV=Mean corpuscular volume, MCH=Mean corpuscular hemoglobin, MCHC=Mean corpuscular hemoglobin concentration, RDW=Red cell distribution width, PLT=Platelets, PCT=Procalcitonin, MPV=Mean platelet volume, ALB=Albumin, ALP=Alkaline phosphatase, AST=Aspartate aminotransferase, GGT=Gamma glutamyl transferase, TP=Total protein, GLO=Globulin, BUN=Blood urea nitrogen, CK=Creatine kinase, Ca=Calcium, K=Potassium, Na=Sodium, Glu=glucose, TBIL=Total bilirubin, CRE=Creatinine.

**Table-4:** Mean±SEM of blood gases and minerals analysis in horses before and after cupping.

Parameter	Before cupping	After 2 weeks
pH	7.42±0.02	7.39±0.01
pCO_2_ (mm Hg)	41.72±0.74	38.78±0.75*
pO_2_ (mm Hg)	94±4.17	102±1.16*
Na (mmpl/l)	139.4±2.66	133.3±1.69
K (mmol/l)	4.24±0.13	3.79±0.11
Ca (mmol/l)	1.61±0.17	1.47±0.13

NS>0.05 and

* <0.05, SEM=Standard error of the mean, Ca=Calcium, K=Potassium, Na=Sodium

## Discussion

This research indicates the influence of wet cupping therapy on the hematological and biochemical parameters in the Arabian horses. In this study, the changes in some of the hematological parameters in Arabian horses before and after cupping are statistically significant. However, as shown in Table-1, a significant increase (after 2-4 weeks) was observed in WBCs which agreed with Al-Kazazz *et al*. [14], Ahmed *et al*. [15], and Mahdavi *et al*. [16], who stated that WBCs will increase after 2 weeks of cupping. However, the other obtained results for the hematological parameters disagreed with Mahdavi *et al*. [16], who mentioned that the values of neutrophils (NEU)%, eosinophils, and MCV were increased after 2 weeks of cupping, and there is a significant decrease in the percentage of LYM, monocytes (MONO), RBCs, HGB, and HCT in the human venous blood. Similar results for non-significantly changes in hematological levels after cupping were reported by Abdullah *et al*. [17]. The selective increase in mononuclear cells including LYM and MONO could be due to selective stimulation of distinct hematopoietic precursor cells in the bone marrow. Further studies are needed to confirm that.

In harmony with the results reported by Al-Kazazz *et al*. [14], the values of ALB as mentioned in Table-2 were observed to be the same before and 2 weeks after cupping. Serum ALP in the current study was found to be increased after the cupping therapy; however, this difference is not considered as statistically significant. This finding disagrees with Mashlool *et al*. [18]. The authors reported that serum ALP levels were significantly decreased after the cupping therapy in humans. AST levels were observed to be non-significantly increased after 3 days after cupping. This increase was followed by decrease in its levels after 2, 4, and 8 weeks of cupping. Comparable results were reported by Mashlool *et al*. [18]. However, AST can normally be detected in many different tissues such as the muscles, liver, kidney, brain, and heart, and its levels are increased if any damage occurs to one of these tissues; therefore, it is not considered as a specific indicator for mammalian liver profiles [19]. However, TP showed a significant increase after 2 and 4 weeks of cupping which disagreed with Al-Kazazz *et al*. [14], who mentioned that there are no change in TP levels before and 2 weeks after cupping in human. TP can be used as indicators to any disturbance in the liver functions or low dietary proteins [20]. GLOB levels were significantly increased after the application of cupping therapy by 3 days and up to 8 weeks of cupping, which is positive for immunosystem and could be explained by the importance of GLOBs as specific groups of proteins that are produced in response to inflammatory stimuli [21]. There was a significant change but within the normal range in the levels of Na (decrease) and K (increase) only 2 weeks after cupping. Similar results for the significant decrease of Na are reported by Refaat *et al*. [22]. In addition, the results of potassium in the present study were in contradictory with Refaat *et al*. [22], who reported the increased levels of potassium after cupping in human. For serum Glu, the obtained results showed a significant increase after 2 and 4 weeks of cupping. These results disagree with Al Showafi [23] and Mashlool *et al*. [18] in which they stated that the Glu levels were lower after applying the cupping therapy than their levels before cupping in human. The cupping therapy can increase the sensitivity of insulin which decreases its levels in the blood of diabetic patients; however, the adverse effects can be attributed to the health status of the animals and some species variations [18]. According to the obtained results that are listed in Table-3, a significant decrease was found in the total WBCs in the samples that were obtained from the cupping blood when compared to the venous blood which is agreed with Mahdavi *et al*. [16]. Sheykhu [24] also reported that WBCs count in cupping blood samples is one-tenth of their count in the venous blood samples. The results of the current study are in agreement for some other hematological parameters such as lower values of MONO%, NEU%, and MCH and higher values of PLT in the cupping blood samples in humans comparing to its values in venous blood, which reported by Mahdavi *et al*. [16]. The significant increase in the PLT values in the cupping blood when compared to its levels in the venous blood is caused by the pressure of sucking which results in further discharge in PLT that has low density which also increases the time that is required for blood clotting which finally leads to the increase of blood flowing and the increase in the end oxygenation of organs [25]. In the current study, significantly higher LYM% and significantly lower BASO% were observed in the cupping blood sample. These results agreed with Al-Kazazz *et al*. [14]. For the biochemical parameters, significantly higher ALP and CK levels were observed. ALP has an important role in the metabolism by permitting the cells to uptake the inorganic form of phosphate [18]. On the other hand, significantly lower values were observed in GGT, GLOB, TBIL, CRE, and Na in the cupping blood when compared to the venous blood. GGT, GLOB, and TBIL are usually used in the assessment of liver function. The current study also disagreed with Mahdavi *et al*. [16] in which lower levels of AST were observed in the cupping blood which can be attributed to the difference between the different species; however, this decrease is not statistically significant. According to the obtained results that are listed in Table-4 for the blood gases analysis before and after the application of cupping therapy, a significant increase in pO_2_ and a significant decrease in pCO_2_ were observed which also proves that cupping therapy increases the blood flow to the organs which leads to better oxygenation [25]. The results of the current study for cortisol concentration before and after cupping as showed in Figure-2 are in agreement with Lin *et al*. [26], who reported a reduction of the cortisol after cupping in people suffered from chronic pain. However, Kim *et al*. [1] suggested that the decrease in cortisol levels is good for pain reduction [26].

## Conclusion

Cupping induced minor changes on the hematological and biochemical parameters in Arabian horses. This is the first trial on the effects of wet cupping therapy on the different parameters in Arabian horses, which would be useful for further investigations on the role of complementary therapies in horses. Our further studies will include different disease models.

## Authors’ Contributions

The fieldworks were done by TS, WE and MH while JH and SA carried out the laboratory examinations. SA and TS wrote the manuscript. The manuscript was reviewed and approved by all of the authors.
